# Purinergic signaling in the gut neuro-glial-immune axis: mechanisms of homeostasis, inflammation, and gastrointestinal dysfunction

**DOI:** 10.3389/fgstr.2026.1954794

**Published:** 2026-09-22

**Authors:** Eduardo E. Valdez-Morales, Andrómeda Liñán-Rico, Raquel Guerrero-Alba

**Affiliations:** 1 Departamento de Fisiología y Farmacología, Centro de Ciencias Básicas, Universidad Autónoma de Aguascalientes, Aguascalientes, Mexico; 2 Investigador por México, Secretaría de Ciencia, Humanidades, Tecnología e Innovación (SECIHTI), Mexico City, Mexico; 3 Centro Universitario de Investigaciones Biomédicas, Universidad de Colima, Colima, Mexico

**Keywords:** enteric glia, enteric neurons, immune cells, inflammation, purinergic signaling

## Abstract

The gastrointestinal (GI) tract contains a highly integrated network composed of the enteric nervous system (ENS), epithelial cells, resident immune populations, and intestinal microbiota. Communication among these components is essential for maintaining intestinal homeostasis and coordinating responses to environmental and inflammatory stimuli. Purinergic signaling regulates synaptic transmission, neuronal excitability, enteric neuron-glia communication, intestinal motility, and epithelial secretion. In parallel, purinergic receptors expressed by epithelial and immune cells modulate barrier integrity, cytokine production, leukocyte recruitment, immune tolerance, and tissue repair. Growing evidence indicates that dysregulated purinergic pathways contribute to neuroinflammation, enteric neuronal loss, intestinal dysmotility, visceral hypersensitivity, and inflammatory bowel disease (IBD). Among purinergic receptors, P2X7 has been strongly implicated in neuroimmune inflammatory responses, particularly in experimental models of IBD, whereas adenosine receptors generally exert immunomodulatory and tissue-protective effects, although these actions are highly context- and cell-dependent. This review summarizes current knowledge on purinergic signaling within the intestinal neuro-glial-immune network, highlighting its roles in gastrointestinal homeostasis and disease. We also discuss emerging therapeutic opportunities targeting purinergic receptors and ectonucleotidases to restore intestinal function and limit chronic inflammation.

## 1 Introduction

The mammalian gastrointestinal (GI) tract contains a diverse microbiome, a highly specialized immune system, and a complex enteric nervous system (ENS). Dynamic, reciprocal interactions among these components are essential for maintaining GI homeostasis (1, 2). Communication between the immune system and ENS is mediated by a wide range of signaling molecules, including classical neurotransmitters such as acetylcholine (ACh) and serotonin (5-HT); neuropeptides such as substance P, calcitonin gene-related peptide (CGRP), and vasoactive intestinal peptide (VIP); as well as cytokines and other mediators, including histamine, prostaglandins, leukotrienes, nucleosides (e.g., adenosine), and nucleotides (e.g., ATP, ADP) (3, 4).

The purinergic system has received increasing scientific attention for nearly a century; however, it became established as a fundamental signaling system in the 1970s, when Geoffrey Burnstock defined the concept of “purinergic transmission” (5). Although initially met with skepticism, interest in this field has grown substantially in recent decades, and it now has a recognized role in physiological and pathological conditions (6).

ATP is a high-abundance intracellular energy molecule (1–10 mmol/L) (7). Purinergic signaling begins with ATP release into the extracellular space via multiple mechanisms, including constitutive or regulated exocytosis, volume-regulated anion channels, maxi-anion channels, ATP-binding cassette transporters, connexins, and pannexins (8–10). Under physiological conditions, eATP is typically present at nanomolar concentrations and contributes to maintaining mucosal barrier integrity and immune balance (11). However, under pathological conditions such as infection, ischemia, inflammation, necrosis, or apoptosis, ATP is released uncontrollably, reaching higher concentrations (6, 12–18). In this condition, eATP acts as a danger-associated molecular pattern (DAMP), triggering acute or chronic inflammatory responses by recruiting and activating immune cells such as T cells, macrophages, and mast cells (19).

Once in the extracellular space, ATP is rapidly hydrolyzed by ectonucleotidases. The ectonucleoside triphosphate diphosphohydrolases (NTPDases) degrade ATP into ADP and subsequently into adenosine monophosphate (AMP), which is further hydrolyzed by the ecto-5’-nucleotidase (NT5E or CD73) to generate adenosine (20). Finally, cells take up adenosine through nucleoside transporters or metabolize it into AMP via adenosine kinase (ADK) or into inosine via adenosine deaminase (ADA) (21).

Extracellular ATP (eATP) was initially identified as a co-neurotransmitter in the ENS, released from intrinsic enteric neurons, sympathetic nerves, and sensory-motor neurons. Experimental studies indicate that eATP contributes to the regulation of intestinal motility, secretion, and sensory functions (22). In addition to neuronal sources, immune cells, mucosal epithelial cells, and commensal bacteria residing in the gut lumen release ATP (23, 24). Once in the extracellular space, it activates P2 purinergic receptors (P2X and P2Y) and is degraded by the ectonucleotidases NTPDase1 (CD39) and CD73, which hydrolyze ATP to ADP and AMP and subsequently generate adenosine, which activates P1 receptors. The balance between eATP and adenosine in the GI tract is associated with maintaining homeostasis (25).

## 2 Purinergic receptors

P1 receptors, also known as adenosine receptors, are G protein-coupled receptors activated by adenosine, and include four subtypes: A1, A2A, A2B, and A3. A1 and A3 receptors are primarily coupled to Gαi/o proteins, inhibiting adenylate cyclase and reducing cyclic adenosine monophosphate (cAMP) levels, whereas A2A and A2B receptors are coupled to Gαs or Gq/11 proteins, stimulating adenylate cyclase or phospholipase C (PLC) activity, respectively (26). These receptors have different affinities for adenosine: A1 and A2A show high affinity (10–30 nM), A3 has lower affinity (100 nM), and A2B requires higher concentrations (> 1 μM) (27).

P2 receptors are divided into ionotropic P2X receptors and metabotropic P2Y receptors. P2X receptors are ATP-gated ion channels permeable to Na^+^, K^+,^ and Ca^2+^. Seven mammalian subunits (P2X1-P2X7) have been identified, which assemble as trimeric structures forming homomeric or heteromeric channels. These receptors display distinct affinities for ATP ranging from nanomolar (P2X1, P2X3) to tens of micromolar (P2X2, P2X4) and hundreds of micromolar (P2X7; EC50 > 100 μM), and varying desensitization kinetics, ranging from fast (milliseconds in P2X1 and P2X3) to slow (seconds in P2X2 and P2X4) to a complete lack of desensitization (P2X7) (28).

P2Y receptors are G protein-coupled receptors activated by extracellular nucleotides, such as ATP, ADP, UTP (Uridine 5’-triphosphate), and UDP (Uridine 5’-diphosphate). Eight mammalian receptors have been described. P2Y1, P2Y2, P2Y4, P2Y6, and P2Y11 couple to Gq/11 proteins, activating PLC, producing inositol 1,4,5-triphosphate (IP_3_), and mobilizing intracellular Ca^2+^. P2Y11 can also couple to Gαs proteins, stimulating adenylate cyclase. Meanwhile, P2Y12, P2Y13, and P2Y14 receptors are coupled to Gαi proteins (29). P2Y receptors have variable affinities for nucleotides, ranging from nanomolar to micromolar (30).

**Table 1** summarizes the main purinergic receptor subtypes expressed throughout the gut, their endogenous agonists, intestinal localization, physiological functions, and pathophysiological relevance.

**Table 1:** Purinergic receptors in the intestinal neuro–glial–immune axis.

Receptor	Principal agonist(s)	Intestinal localization	Physiological function	Pathophysiological relevance	Ref.
A1	Adenosine	ECs, submucosal neurons, smooth muscle	Inhibits 5-HT release; suppresses Cl^−^ secretion; reduces motility	Antispasmodic protection; modulation of hypersecretion	(31, 32)
A2A	Adenosine	Inhibitory neurons, immune cells	Stimulates NO synthesis; immunomodulation	Anti-inflammatory; regulates neuroinflammation	(33)
A2B	Adenosine (high concentrations)	Epithelium, inhibitory motoneurons, myenteric glia	Stimulates Cl^−^ secretion; modulates permeability and colonic motility; regulates IL-1β, GDNF, and substance P release from EGC	Context-dependent: Proinflammatory under chronic inflammation; glial immunomodulation	(34–38)
A3	Adenosine	ECs, epithelium	Inhibits 5-HT release	Dual effects: protection vs inflammation	(31, 39)
P2X1	ATP, α,β-meATP	ECs	Autoinhibition of 5-HT release	Fine sensory regulation	(40)
P2X2	ATP	IPANs, NOS+ motoneurons, secretomotor neurons, EGC	Fast EPSPs; modulation of peristalsis	Knockout alters ileal transit; promotes inflammation	(41–43)
P2X3	ATP, α,β-meATP	IPANs, interneurons, submucosal neurons	Mechanosensation; peristaltic reflex	Visceral hypersensitivity; impaired motility	(41, 42, 44)
P2X4	ATP; ivermectin (positive modulator)	Enterocytes, Paneth, goblet, enteroendocrine cells; neurons	Epithelial secretion; synaptic modulation; luminal ATP sensing	Inflammation; visceral pain; epithelial dysfunction	(45–47)
P2X5	ATP	Myenteric neurons	Poorly defined	Possible excitatory role	(48)
P2X6	ATP	Enteroendocrine cells	Poorly defined	Hormonal signaling modulation	(46)
P2X7	BzATP (high potency), ATP (high concentrations)	Neurons, glia, epithelium, macrophages	Inflammation; neuronal death; GLUT2 regulation	Neuroinflammation; dysmotility; IBD	(49–54)
P2Y1	ADP; ATP (via conversion)	NOS+ interneurons, EGCs, PDGFRα cells	Slow EPSPs; glial Ca²^+^ signaling; inhibitory junction potentials	Altered motor reflexes; glial amplification	(52, 55–59)
P2Y2	ATP, UTP	Epithelium, VIP+ neurons, immune cells	Cl^−^/K^+^ secretion; inhibits Na^+^ absorption; bicarbonate secretion	Diarrhea; epithelial repair	(60–64)
P2Y4	UTP	Epithelium	Cl^−^/K^+^ secretion; stimulates 5-HT release	Epithelial homeostasis	(61, 62, 65)
P2Y6	UDP	Epithelium, interneurons	NaCl secretion; modulation of excitatory/inhibitory pathways	Inflammatory response; motility regulation	(66, 67)
P2Y11	ATP	Immune cells	Increases Ca²^+^ and cAMP	Regulation of systemic inflammation	(29)
P2Y12	ADP	IPANs, muscularis macrophages	Inhibits adenylate cyclase; neuronal modulation	Neuroimmune regulation	(67, 68)
P2Y13	ADP	Immune cells	Gi-coupled signaling	Immune regulation	(68)
P2Y14	UDP-glucose, UDP	Immune cells, epithelium	Chemotaxis; innate defense	Mucosal inflammation	(68)

5HT, serotonin (5-hydroxytryptamine); ADP, adenosine diphosphate; AMP, adenosine monophosphate; ATP, adenosine triphosphate; BzATP, benzoylbenzoyl-ATP; cAMP: cyclic adenosine monophosphate; ECs, enterochromaffin cells; EGCs, enteric Glial cells; EPSPs, excitatory postsynaptic potentials; GLUT2, glucose transporter type 2; IBD, inflammatory bowel disease; IPANs, intrinsic primary afferent neurons; NO, nitric oxide; NOS, nitric oxide synthase; PDGFRα, platelet-derived growth factor receptor-alpha; UDP, uridine diphosphate; UDP-glucose, uridine diphosphate glucose; UTP, uridine triphosphate; VIP, vasoactive intestinal peptide.

## 3 Functional organization of the ENS

The ENS is a complex neural network of ganglia embedded within the GI tract of mammals. It comprises approximately 200–600 million neurons, along with a larger population of enteric glial cells (EGCs). These cells are organized into two interconnected plexuses: the submucosal or Meissner’s plexus, located in the submucosal layer of small and large intestines, and involved in regulating secretion, absorption and local blood flow functions, and the myenteric or Auerbach’s plexus located between the longitudinal and circular smooth muscle layers (muscularis externa) of esophagus to the rectum, and its main function is to control GI motility (69).

Enteric neurons are functionally classified into intrinsic primary afferent neurons (IPANs), interneurons, and motoneurons. IPANs constitute about half of the neurons in the myenteric plexus and 10-15% of the submucosal plexus. These neurons are activated by enterochromaffin (EC) cells, which transduce chemical and mechanical stimuli within the intestinal lumen. IPANs initiate intrinsic reflexes, including peristalsis and secretomotor response, by establishing synapses with interneurons, which in turn transmit signals along ascending and descending pathways within the ENS circuitry (70–72). Motoneurons are the final output of these circuits and are subdivided into: excitatory and inhibitory motoneurons that innervate the muscularis externa and regulate smooth muscle contractions; vasomotor neurons that regulate blood flow; secretomotor neurons that regulate the secretion of chloride and water; and motor neurons that innervate enteroendocrine cells and modulate immune and defensive functions (73).

The neurochemical coding of enteric neurons is highly diverse and varies across species and GI tract regions. Recent single-cell RNA sequencing studies have identified at least 22 distinct neuronal subtypes, each characterized by a unique gene expression profile (74–77). Although these molecular classifications provide valuable insights, the precise functional roles of many neuronal subtypes remain incompletely defined. Nevertheless, general associations between neurotransmitters and neuronal function have been established. For instance, ACh is the predominant neurotransmitter in most excitatory myenteric neurons, whereas inhibitory motoneurons primarily use nitric oxide (NO), often in combination with VIP, ATP, neuropeptide Y (NPY), and enkephalin. Noncholinergic secretomotor and vasomotor neurons use VIP as the main neurotransmitter (78–80). Furthermore, substance P or a related tachykinin is also generally found in excitatory motoneurons, and ATP has been identified as a secondary neurotransmitter in the descending interneurons (81).

In addition to neurons, the ENS contains a large population of EGCs, whose numbers exceed those of neurons by 6–7 times in the myenteric plexus and approximately twice in the submucosal plexus of the human intestine (82). These cells have a star-shaped morphology, like astrocytes, and express the classical glial markers, glial fibrillary acidic protein (GFAP) and Ca^2+^-binding protein S100β. EGCs are distributed throughout the gut wall, including the muscular externa, along axons within or between ganglia of the ENS, the lamina propria, and in close association with epithelial cells (83). For a long time, EGCs were considered solely to provide structural and metabolic support for neurons; however, they are now recognized as key regulators of neuronal activity, synaptic transmission, and reflex control of intestinal motility through close interaction with enteric neurons (84–86). Moreover, they actively participate in immune responses by expressing cytokines, chemokines, and major histocompatibility complex (MHC) class II molecules (87). Bidirectional communication between EGCs and immune cells contributes to maintaining the intestinal epithelial barrier integrity, host defense, and modulation of inflammatory responses (88).

The coordinated interaction among enteric neurons, EGCs, and immune cells forms a functional neuro-glial-immune network that is essential for GI homeostasis. Disruption of this network can lead to neuroinflammation, altered motility, and long-term GI dysfunction (87, 89).

### 3.1 Purinergic neurotransmission in the ENS and its role in GI motility

Purinergic neurotransmission plays a role in regulating GI motility within the ENS. ATP is released as a co-transmitter from sympathetic nerves together with noradrenaline (90), and from enteric inhibitory motoneurons along with VIP or NO (91). The molecular interactions among these inhibitory neurotransmitters remain unclear.

ATP is considered an inhibitory neurotransmitter because it induces inhibitory junction potentials (IJPs) by activating P2Y1 receptors on PDGFRα (Platelet-derived growth factor receptor alpha) cells. These cells are electrically coupled to smooth muscle cells, resulting in muscle relaxation (92). In addition, ATP mediates synaptic transmission between enteric neurons, generating fast and slow excitatory postsynaptic potentials (EPSPs) (59, 93).

Adenosine inhibits intestinal motility either directly, through activation of A1 receptors on smooth muscle, or indirectly, by inhibiting cholinergic or tachykinergic transmission from enteric neurons. A2A receptors exert their inhibitory effects via NO synthesis in inhibitory motoneurons (33). The role of A2B and A3 receptors in intestinal motor control remains poorly understood. A2B receptors are expressed on NO-releasing enteric inhibitory motoneurons in the distal colon of mice and may contribute to intestinal relaxation (34). Whereas A3 receptors are expressed in the myenteric ganglia of the distal colon in rats, where their contribution to gut motility remains unclear (39). In the human submucous plexus (hSMP), however, A3 receptors mediate a tonic inhibitory modulation of synaptic transmission by endogenous adenosine (94), suggesting that their contribution may be more relevant to enteric neurotransmission than to motor control per se.

Fast EPSPs mediated by ATP contribute to excitatory neurotransmission in ascending and descending reflex pathways and are generated through activation of P2X receptors in enteric neurons (95). Multiple P2X receptor subunits have been detected in enteric neurons across different species, including P2X1, P2X2, P2X3, P2X4, P2X5, and P2X7 subunits (42, 47, 48, 53, 94, 96, 97). In the human submucous plexus, transcripts for P2X1–P2X7 have been detected, and P2X1, P2X2, and P2X3 immunoreactivity has been localized to HuC/D-positive neurons, with P2X2 being the most widely expressed subunit, followed by P2X1 and, less frequently, P2X3. Notably, P2X1 is highly expressed and frequently co-localized with P2X2 in human submucosal neurons, a feature not previously reported in other species, and selective P2X antagonists block synaptic transmission, supporting a functional role for these channels in human enteric neurotransmission (94). Additional studies showed P2X4 mRNA expression in 90% of myenteric neurons using single-cell RT-PCR, along with strong immunoreactivity in cultured mouse myenteric neurons (47), although P2X4 immunoreactivity is not evident in intact ganglia (98). P2X7 expression has been demonstrated in primary cultures of guinea pig myenteric neurons using electrophysiology and single-cell RT-PCR techniques (53). P2X7 immunoreactivity has also been detected in EGCs and myenteric and submucosal ganglia throughout the rat GI tract (54).

The neurochemical identity of the neurons expressing each subunit has been characterized in greater detail in animal models, particularly in the myenteric plexus. In guinea pig myenteric neurons, P2X2 is expressed in approximately 25% of NOS-immunoreactive inhibitory motoneurons, as well as in calbindin-positive IPANs and VIP-positive secretomotor neurons (96). The P2X3 subunit is expressed in a smaller proportion of NOS-immunoreactive motoneurons, as well as in calretinin-positive interneurons and submucosal neurons expressing calretinin and NPY, which are mainly intrinsic sensory neurons (42). In mice, 22%, 9%, 6%, and 68% of P2X5-immunoreactive myenteric neurons co-express calretinin, calbindin, NOS, and VIP, respectively (48).

The functional role of P2X receptors in enteric neurotransmission has been demonstrated in several studies. P2X receptors modulate ACh release presynaptically at nerve terminals innervating longitudinal smooth muscle in the small intestine of guinea pigs and mice (99–101), and regulate synaptic strength postsynaptically through crosstalk with GABA_A_, nicotinic ACh, and 5-HT_3_ receptors in myenteric and submucosal neurons (102–104). Genetic studies in mice provide evidence for the functional role of P2X receptor subtypes in intestinal motility. Deletion of the P2X2 subunit gene abolishes ATP-induced fast EPSPs in inhibitory motoneurons and is associated with alterations in ileal peristalsis, supporting a role for the P2X2 receptor in enteric neurotransmission and intestinal motor control (105). Similarly, P2X3 deletion alters ileal peristalsis (41), consistent with the contribution of P2X3-expressing intrinsic sensory neurons to the regulation of the peristaltic reflex.

Anatomical and pharmacological studies corroborate that P2X3-expressing intrinsic sensory neurons project into the submucosa and form connections with interneurons and motoneurons in the myenteric and submucosal plexuses. Furthermore, ATP and α,β-meATP, a selective agonist for P2X1, P2X3, and P2X2/3 receptors, activate submucosal terminals of intrinsic sensory neurons, supporting the hypothesis that ATP released from mucosal epithelial cells acts on P2X3 receptors to detect luminal distension and initiate the peristaltic reflex (44). Functional studies in the rat colon suggest that P2X7 contributes marginally to regulating longitudinal muscle motility under basal conditions; conversely, the inflammation-associated increase in P2X7 expression is linked to altered colonic motility (49), suggesting that P2X7 signaling contributes to inflammation-associated colonic dysmotility.

Whereas P2X channels mediate fast excitatory transmission, metabotropic P2Y receptors are responsible for slower, modulatory responses in the ENS. Among them, P2Y1 is the best characterized. Immunohistochemical studies localized P2Y1 in NOS-positive myenteric neurons in mice and guinea pigs (106, 107). Functional electrophysiological studies in guinea pig preparations further show that electrical stimulation of mucosal nerve endings induces slow EPSPs in myenteric neurons that are also sensitive to the P2Y1-selective antagonist, MRS2179 (56). Similarly, pharmacological studies show that MRS2179 inhibits ATP-evoked slow EPSPs in submucosal secretomotor neurons (57, 58). Furthermore, guinea pig P2Y1 receptors in descending myenteric interneurons respond to mucosal electrical stimulation with slow EPSPs that are inhibited by MRS2179, suggesting a role for P2Y1 receptors in the transmission between descending interneurons during inhibitory reflexes (59).

Other P2Y receptors have been identified in ENS; the P2Y2 and P2Y12 receptors have been identified in NOS-positive myenteric neurons in the rat distal colon (68). In guinea pigs, most P2Y2-immunoreactive myenteric neurons co-express calretinin and VIP, and 40-60% co-express P2X3; in the submucosal plexus, all P2X3-positive neurons also express P2Y2 (64). Approximately 30% of guinea pig myenteric neurons express P2Y6, with 28–35% co-expressing NOS (inhibitory motoneurons and descending interneurons) and 41-47% with calretinin (excitatory motoneurons and ascending interneurons). Additionally, about 40% of neurons in the myenteric and submucosal plexuses exhibit P2Y12 immunoreactivity, all of which are calbindin-positive, indicating predominant expression in IPANs (67). These expression patterns are consistent with localization within both inhibitory and excitatory motoneuron and sensory neuron populations, although their specific functional contributions to gut motility remain unclear.

Purinergic signaling is one of the most widely characterized mechanisms of neuron-glia communication involved in the regulation of gut motility. Pharmacological studies demonstrate that stimulation of intrinsic and extrinsic (sympathetic) neurons induces the release of ATP and ADP, which activates glial P2Y1 and P2Y4 receptors and increases intracellular Ca^2+^ through PLC-dependent signaling (55, 108). More recent evidence shows that stimulation of a single enteric neuron can induce ATP release through pannexin channels, activating 2 to 3 adjacent EGCs, indicating that neuron-glia communication is highly localized (109). Furthermore, activation of the glial P2Y1 receptors can induce ATP release via connexin-43 (Cx43) hemichannels (110), suggesting that interglial signaling may amplify neuronally initiated responses.

Under intestinal inflammatory conditions, a bidirectional purinergic signaling loop between neurons and EGCs has been described. This loop involves pannexin-dependent ATP release after activation of neuronal P2X7 receptors, which, in turn, stimulates glial P2Y1 receptors. Activated EGCs produce NO, which induces additional ATP release via Cx43 hemichannels, leading to progressive eATP accumulation. Elevated eATP concentrations then reactivate neuronal P2X7 receptors, amplifying the cycle and ultimately triggering neuronal death, which may contribute to motility disorders (52). Supporting this idea, purinergic activation of EGCs enhances activity in ascending motor pathways while suppressing descending pathways, indicating pathway-specific glial modulation of motility (84).

In the mouse submucosal plexus, VIP modulates neuron-glia communication by acting on vasoactive intestinal peptide receptor 1(VPAC1) on cholinergic neurons, promoting purine release that triggers a transient Ca²^+^response in enteric glia. In contrast, activation of VPAC2 receptors inhibits glial activity (111), indicating that additional neurotransmitters regulate ATP release and glial activation. Moreover, glial responses to purines vary across the GI tract. For instance, ADP-induced Ca^2+^ responses in the myenteric plexus differ between the duodenum and colon, with greater heterogeneity in the duodenum and more uniform and robust responses in the colon (112). In addition, ATP-induced Ca^2+^ responses are stronger in intraganglionic glia than in extraganglionic glia (113), adding further complexity.

Overall, purinergic control of GI motility rests on three axes: ATP acting at P2X channels for fast excitatory transmission, P2Y1 signaling along the inhibitory pathway and in neuron–glia communication, and adenosine providing tonic inhibitory modulation. In each case, the functional outcome depends on receptor subtype, cell type, circuit position, anatomical region along the GI tract, and the pathophysiological context. Particularly, in human tissue, these variables remain incompletely characterized.

### 3.2 Purinergic modulation of intestinal epithelial secretion and neuroepithelial crosstalk

GI secretions occur alongside motility, facilitating digestion, nutrient absorption, pH regulation, and waste elimination. Chemical and mechanical stimuli in the GI lumen trigger secretory reflexes involving EC cells, IPANs, interneurons, secretomotor neurons, and mucosal epithelial cells (31).

Advancements in analytical techniques have enabled quantification of ATP concentration in mucosal epithelial cells, revealing micromolar levels within the sensitivity range of purinergic receptors. These cells also release smaller amounts of UTP and adenosine (114). Because of their location and function, intestinal epithelial cells (IECs) are exposed to a wide range of ATP-releasing stimuli, including mechanical, osmotic, and metabolic stress, pathogen-related nucleotide release and cell lysis, as well as hormonal and neuronal signals (115, 116). Commensal gut bacteria also contribute to ATP production and release (23).

The intestinal lumen is continuously exposed to dietary nutrients, pH fluctuations, mechanical forces, osmotic changes, and commensal microbiota. EC cells sense these stimuli and secrete 5-HT (117). 5-HT acts on IPANs, triggering action potentials and inducing neurotransmitter release at synapses with interneurons and secretomotor neurons, thereby initiating secretory and peristaltic reflexes (118). Studies using BON cells, a human carcinoid cell line that secretes 5-HT and is widely used as a human EC cell model, have provided evidence for the functional involvement of purinergic receptors in 5-HT release. In BON cells, endogenous adenosine has been reported to inhibit 5-HT release by activating A1 and A3 receptors (31). Subsequent studies demonstrated that ATP exerts autocrine inhibition of 5-HT release in a subset (<20%) of BON cells in intact mucosa, likely through activation of P2X1 or P2X3 receptors (40). In addition, UTP stimulates 5-HT release, possibly via P2Y4 and P2Y6 receptors in this EC cell model (65). Additional evidence indicates that P2Y1 receptor activation promotes 5-HT release through the Gαq/PLC/IP3 signaling pathway, whereas P2Y12 receptor activation inhibits secretion via Gαi-mediated inhibition of adenylate cyclase (118). These findings support a role for purinergic signaling in the regulation of EC cell function; however, because these observations were obtained in the BON cell line, their relevance to primary human EC cells and intestinal physiology *in vivo* remains to be established.

Electrophysiological and pharmacological studies performed in different experimental systems indicate that purinergic receptors regulate intestinal epithelial secretion in a cell- and species-dependent manner. In the human intestinal epithelial T84 cell line, pharmacological A2B receptor activation on the apical membrane stimulates chloride secretion (38). In contrast, pharmacological activation of A1 receptors on guinea pig submucosal neurons inhibits chloride secretion (32), while mechanical stimulation induces nucleotide release and a P2Y1-dependent neural reflex that promotes chloride secretion in the distal colon (119).

Genetic deletion of P2Y2 and P2Y4 in mice reduced intestinal chloride secretion by 40% and 60%, respectively, compared with the wild-type mice (61). In mice, further pharmacological activation of P2Y2 and P2Y4 receptors on the luminal membrane of IECs induced potassium secretion in the distal colon (62), whereas P2Y2 receptor activation at the apical membrane inhibited sodium absorption (63). P2Y2 receptor activation also contributes to duodenal bicarbonate secretion by inducing intracellular Ca^2+^ release and extracellular Ca^2+^ entry via store-operated channels (60). In rat colonic IECs, basolateral P2Y6 receptor activation induced NaCl secretion through a synergistic increase in intracellular Ca^2+^ and cAMP (66). Collectively, these findings provide functional evidence that purinergic signaling regulates intestinal ion transport, but the underlying responses are dependent on receptor subtype, cellular localization, and species, and findings from cell lines or animal models should not be directly extrapolated to human intestinal physiology.

A recent study examined P2X receptor expression in human small intestinal and colonic tissue using single-cell RNA sequencing (RNA-seq). They found robust mRNA expression of the P2X4 receptor in the small intestine, where it was detected in proximal enterocytes, goblet cells, and Paneth cells. In the colon, P2X4 is strongly expressed across multiple epithelial cell types, including undifferentiated cells, distal enterocytes, goblet cells, Paneth cells, and enteroendocrine cells. Enteroendocrine cells express high levels of P2X6, whereas P2X1, P2X5, and P2X7 are expressed at very low levels (46). These findings support differential P2X receptor expression across human intestinal epithelial cell populations; however, the functional contribution of individual receptors to epithelial secretion and absorption has been poorly studied.

Functional evidence for P2X4 and P2X7 signaling in epithelial ion transport has been obtained in rat colonic preparations. P2X4 and P2X7 expression was detected using RT-PCR and immunohistochemistry, predominantly in the upper crypt regions and submucosal secretomotor neurons. In colonic mucosa-submucosal preparations, exogenous applications of ATP and BzATP induced transepithelial currents. BzATP responses on the serosal side were partially blocked by TTX, indicating the involvement of submucosal secretomotor neurons, whereas mucosal application induced TTX-insensitive transepithelial currents, consistent with a direct epithelial component. Moreover, pharmacological inhibition with the P2X4 antagonist 5-BDBD and the P2X7 antagonist AZ10606120 significantly reduced ATP- and BzATP-induced responses, providing pharmacological evidence for the involvement of P2X4 and P2X7 in regulating colonic epithelial ion transport in rats (45).

P2X7 signaling has also been implicated in intestinal glucose absorption. In the rat jejunum, pharmacological activation of the P2X7 receptor was reported to reduce luminal GLUT2 expression in enterocytes, suggesting a role for P2X7 in regulating glucose transport (51). Consistent with this finding, P2X7-deficient mice exhibit increased membrane GLUT2 expression in enterocytes, accompanied by elevated blood glucose and serum insulin levels (50), providing complementary genetic evidence for P2X7’s role in intestinal glucose transport. Together, these studies support a functional contribution of P2X4 and P2X7 to epithelial transport; however, the evidence derived predominantly from rodent models, and the extent to which these findings apply to human intestinal physiology remains to be determined.

## 4 Organization of the intestinal mucosal immune system

The mucosal immune system plays a pivotal role in tolerating dietary antigens and distinguishing between beneficial commensal microorganisms and pathogens, thereby maintaining GI homeostasis. This system comprises the epithelial barrier, the lamina propria, and the gut-associated lymphoid tissue (GALT). GALT is further organized in Peyer’s patches, isolated lymphoid follicles (ILFs), and mesenteric lymphoid nodes (MLNs), which together contain the largest population of immune cells in the body (120).

The intestinal epithelial barrier is a complex and dynamic structure formed by a monolayer of specialized epithelial cells joined by tight junctions and overlaid by mucus. This barrier separates the intestinal lumen from the lamina propria, allowing nutrient absorption while restricting pathogen and toxin entry (121). Differentiated IECs include enterocytes, enteroendocrine cells, goblet cells, Paneth cells, and microfold cells (M cells), each with a distinct function in maintaining barrier integrity. Enterocytes are the most abundant IECs and specialize in absorbing nutrients, electrolytes, and water (122). Enteroendocrine cells function as sensory cells that secrete hormones, such as 5-HT, glucose-dependent insulinotropic peptide (GIP), cholecystokinin (CCK), glucagon-like peptide-1 (GLP-1), and peptide YY (PYY), in response to luminal stimuli; they play a role in GI motility, nutrient sensing, glucose metabolism, and regulation of intestinal permeability (123, 124). Goblet cells contribute to barrier function by secreting bioactive molecules, such as mucins (MUC2), trefoil peptides (TFF3), and resistin-like molecule *β* (RELM*β*), which are involved in epithelial restitution and mucosal repair (125). Paneth cells located at the base of Lieberkühn’s crypts secrete antimicrobial proteins, such as defensins, cathelicidins, secretory phospholipase A2 (sPLA2), and lysozymes, providing protection against pathogens (126). M cells are located within GALT and specialize in phagocytosing and transcytosing luminal antigens and pathogens to the basolateral side, where they interact with antigen-presenting cells, such as macrophages and dendritic cells, representing a critical initial step in mucosal immune responses (127). Furthermore, the intestinal epithelium contains intraepithelial lymphocytes (IELs) that are interspersed between epithelial cells along the lateral intercellular space and basolateral membrane. IELs exhibit both innate and adaptive immune features and represent one of the most abundant T-cell populations in barrier tissues, serving as a first line of defense against invading pathogens (128).

The lamina propria is a thin layer of loose connective tissue located beneath the epithelium and, together with the epithelium and basement membrane, forms the mucosa. It is the intestine’s main immunological compartment, containing most intestinal immune cells, as well as blood and lymphatic vessels and mucosal innervation. Immune cell populations in the lamina propria include lymphocytes, predominantly CD4^+^ T cells, as well as cytotoxic CD8^+^ T cells, regulatory T cells (Treg), and plasma cells derived from B lymphocytes that mainly produce polymeric immunoglobulin A (pIgA). The lamina propria also contains a broad array of innate immune cells, including innate lymphoid cells (ILCs), eosinophils, dendritic cells, macrophages, and mast cells (120).

GALT is primarily located within the lamina propria, although larger structures, such as Peyer’s patches, can extend into the submucosa (129). Peyer’s patches are distributed throughout the small intestine, with increasing density toward the terminal ileum, where they form a lymphoid ring at the ileocecal junction. These structures serve as sites for mucosal adaptive immunity, particularly the IgA-mediated responses. The luminal surface of Peyer’s patches is covered by follicle-associated epithelium (FAE), which has a reduced mucus layer that facilitates antigen exposure and is enriched in M cells. Beneath the FAE lies the subepithelial dome (SED), a specialized region predominantly composed of dendritic cells, along with macrophages, CD4^+^ T cells, and B cells producing IgA, IgM, and IgG. Through this organization, M cells transfer antigens to dendritic cells, which subsequently present them to CD4^+^ T cells and B cells in Peyer’s patches (130). Alternatively, antigen or antigen-loaded dendritic cells can migrate via lymphatic drainage to MLNs, where they are presented to naïve T cells (131). ILFs are distributed throughout the intestine and serve as sites for T-cell-independent IgA production. In humans, two types have been described: mucosal ILFs (M-ILFs) located in the lamina propria of the ileum and distal colon, and submucosal ILFs (SM-ILFs) primarily found in the colon (129, 132).

In addition to the immune cells in the mucosa, the muscularis externa contains abundant immune cells. Macrophages are the predominant immune cell in the muscularis externa of the ileum and colon, where they are referred to as muscularis macrophages. They coexist with a heterogeneous population of immune cells, including dendritic cells, ILCs, eosinophils, plasma cells, natural killer (NK) cells, and T and B cells (133). Muscularis macrophages comprise resident and infiltrating subpopulations. Resident muscularis macrophages express CD163 and regulate intestinal smooth muscle contraction by secreting bone morphogenetic protein 2 (BMP2), which activates BMP receptors on enteric neurons (134, 135). Infiltrating macrophages in the muscularis and lamina propria have a limited role in regulating enteric neurons but are implied to contribute to the inflammatory response (136, 137).

## 5 Bidirectional neuroimmune communication in the intestinal mucosa

Enteric neuronal terminal varicosities and EGCs are located in close proximity to lymphoid tissue and resident immune cells within the lamina propria (138). An extensive network of nerve fibers has been identified throughout all compartments of Peyer’s patches, including lymphoid nodes, interfollicular region, FAE, and SED (139). Similarly, peptidergic nerve fibers are found in close apposition to immune cells within the SED of Peyer’s patches and adjacent villi (140, 141). Furthermore, adrenergic, cholinergic, serotoninergic, neuropeptide (such as Substance P and VIP), and purinergic receptors are expressed across multiple immune cell types, indicating that neuronal signals can directly modulate these cells’ function (142–148).

Reciprocally, enteric neurons express pattern recognition receptors, such as Toll-like receptors (TLRs), cytokine receptors including tumor necrosis factor-alpha (TNF-α) and transforming growth factor-β (TGF-β), and lipid mediator receptors. They also produce immune mediators, such as interleukin-6 (IL-6) and IL-18, enabling them to detect and respond to inflammatory signals (149–151). Similarly, EGCs detect immune-derived signals via TLRs and cytokine receptors (e.g., IFN-γ receptors). Upon activation, EGCs secrete cytokines and chemokines implicated in both pro- and anti-inflammatory effects, thereby contributing to the regulation of intestinal barrier integrity, immune responses, host defense, and tissue repair (152).

Soluble mediators, including cytokines, chemokines, and neurotransmitters (e.g., ACh and substance P), play a role in tripartite communication among immune cells, enteric neurons, and EGCs. Recent literature has extensively reviewed this complex interplay (88, 153–156).

### 5.1 Immunomodulatory effects of purinergic signaling

Here, we focus on recent evidence identifying purines as mediators of ENS-immune communication involved in gut functions and dysfunction. Beyond its role as a co-neurotransmitter in the ENS, ATP can be released in response to various inflammatory stimuli. For instance, bacterial lipopolysaccharide (LPS) induces ATP release from macrophages by activating pannexin-1 (Panx1) (18). Infections with intracellular protozoa also release ATP from macrophages and T-cells (13, 17). In a murine colitis model, connexin 43-dependent ATP release in EGCs exacerbates tissue damage by inducing neuroinflammation and contributing to the development of motility disorders (52). In addition, ATP can be released in large amounts after tissue damage or cell death, where it acts as a DAMP recognized by immune cells and activates both innate and adaptive immune responses (6).

#### 5.1.1 P2X receptors in immunoregulatory responses

P2X receptors are widely recognized for their roles in immune regulation, and their expression and function are receiving increasing attention. In mice and humans, P2X1, P2X4, and P2X7 receptors have been detected in multiple immune cell populations, including monocytes, macrophages, neutrophils, eosinophils, mast cells, NK cells, T cells, and B cells (157, 158). In humans, P2X2 expression has also been detected in B cells (156), while P2X5 expression has been reported in T cells (159). However, P2X2 and P2X5 receptors have received comparatively little attention in the immune system, and their functions remain poorly understood. Functional studies indicate that P2X1 and P2X4 receptors are involved in activating human T cells and mast cells (160). In contrast, the P2X7 receptor has been extensively studied in the immune system, with numerous functions identified in regulating immune responses. P2X7 receptor can promote pro-inflammatory immune responses and exacerbate inflammatory diseases through multiple mechanisms: 1) activation of transcription factors that induces the expression of genes involved in inflammation, including NF-κB, nuclear factor of activated T cells (NFAT), and hypoxia-inducible factor 1α (HIF1α); 2) upregulation of pro-inflammatory cytokines, such as TNF-α and IL-6, as well as chemokines including CCL2 and CXCL2, across various immune cells types; and 3) enhancement of neutrophil recruitment in mice (161). The P2X7 receptor is also one of the most potent activators of the NLRP3 inflammasome, inducing IL-1β release in mouse macrophages and neutrophils (162). Furthermore, P2X7 receptor activation is associated with T cell activation in intestinal barrier dysfunction (163). Finally, P2X7 receptor activation can induce cell death, increasing DAMP release and amplifying inflammation (164).

The P2X7 receptor has been implicated in purinergic communication between ENS and immune cells, particularly in intestinal inflammation. P2X7 expression has been detected in enteric neurons, EGCs, and various immune cell types (165). During intestinal inflammation, increased P2X7 mRNA and protein expression has been reported in the intestinal epithelium and lamina propria in both murine models and human intestinal biopsies with IBD, with P2X7 colocalizing with dendritic cells and macrophages (49, 166, 167). Functional evidence from murine experimental colitis models further supports a proinflammatory role for P2X7. In P2X7-deficient mice, genetic loss of the receptor confers protection against chemically induced colitis and reduces disease-associated manifestations (167). Consistently, pharmacological inhibition of P2X7 using the selective antagonist A740003 reduces T cell and macrophage infiltration into the lamina propria, decreases colonic levels of proinflammatory cytokines, such as TNF-α and IL-1β, and ameliorates intestinal inflammation in murine experimental colitis (168). P2X7 signaling has also been implicated in Treg cell death and retention in MLNs, affecting intestinal immune tolerance and contributing to inflammation in chemically induced colitis in mice (169). In parallel, experimental studies indicate that P2X7 receptor signaling may contribute to inflammation-associated alterations in ENS integrity and function (166). However, whether these experimental findings translate directly to therapeutic benefit in human IBD remains uncertain and therefore requires further investigation.

Other P2X receptors have also been implicated in ENS-immune interactions. P2X2 receptor expression has been detected in EGCs from mice and humans, and experimental studies indicate that P2X2 contributes to enteric gliosis and neuroinflammation (43). In murine models of intestinal inflammation induced by surgical manipulation or trauma, increased eATP release activates P2X2 receptors on EGCs and promotes a reactive glial phenotype characterized by increased expression of gliosis-associated markers, including GFAP and Nestin, and the release of IL-6 and CXCL2, which exacerbate neuroinflammation, promote immune cell infiltration, and contribute to postoperative motility disorders (43). P2X4 receptor expression has been detected in enteric neurons, immune cells, and epithelial cells in mice (170). In a mouse model of cecal ligation and puncture-induced sepsis, experimental activation of macrophage P2X4 receptors increased bacterial killing, reduced inflammatory mediator production, and conferred protection against sepsis (171), suggesting a protective role of P2X4 in this context. Conversely, genetic deletion of P2rx4, which encodes the P2X4 receptor subunit, exacerbated dextran sulfate sodium (DSS)-induced colitis and was associated with intestinal dysbiosis and altered intestinal mucosal permeability, whereas pharmacological positive modulation of the P2X4 receptor with ivermectin attenuated inflammation and restored the gut microbiota in wild-type mice (172). In a separate rat model of colitis, treatment with a selective P2X4 antagonist attenuated colitis-associated visceral pain, accompanied by suppression of NLRP3/IL-1β signaling and preservation of tight junction protein expression (173). Altogether, these findings suggest that P2X4 signaling can modulate intestinal inflammation, epithelial barrier integrity, gut microbiota composition, and visceral sensitivity, though its effects depend on the specific cellular and experimental model. Despite these advances, most of the available evidence comes from murine models, while human data are more limited and primarily support receptor expression. Thus, although P2X2 and P2X4 may contribute to ENS-immune interactions in the GI tract, their precise cellular mechanisms and relevance to human intestinal disease remain incompletely defined.

#### 5.1.2 P2Y receptors in immunoregulatory responses

P2Y receptors regulate diverse effects on neuroimmune interactions in the gut, communicating enteric neurons and EGCs with intestinal immune cells. In the mouse myenteric plexus, expression studies indicate that P2Y1 receptors are broadly distributed in enteric neurons and EGCs, whereas P2Y12 receptors are predominantly detected in muscularis macrophages (174). Consistent with these expression patterns, functional studies indicate that ADP increases neuronal and glial activity through P2Y1 receptors, while P2Y12 signaling in muscularis macrophages appears to constrain this response (174). An earlier study reported P2Y12 expression in calbindin-immunoreactive neurons in the guinea pig intestinal myenteric plexus (67), highlighting potential interspecies differences and the need for caution when extrapolating receptor localization and function across experimental models. Thus, current evidence supports a role for P2Y1 and P2Y12 receptors in neuro-glia-macrophage communication, although their cellular distribution and relative contribution may vary among species and experimental settings.

Given their differential effects across species, nucleotide ligands, G-protein coupling, cell type, and immune cell maturation and activation state, P2Y receptors have been implicated in regulating pro- and anti-inflammatory responses in both physiological and pathological conditions. Most studies characterize P2Y2, P2Y6, P2Y12, and P2Y14 as proinflammatory, suggesting that a selective antagonist might reduce inflammatory disease severity. These subtypes can regulate immune cell migration, phagocytosis, cytokine production, antigen presentation, and other inflammatory responses, thereby contributing to communication between immune, epithelial, neuronal and glial cells (172). A recent review emphasizes the broad immunomodulatory functions of P2Y receptors and their relevance to inflammatory processes in infection and autoimmunity (175).

Among the P2Y receptors expressed in the intestinal mucosa, P2Y2 has received particular attention because of its potential involvement in immune cell recruitment. Increased P2Y2 expression has been reported in both colonic tissue from IBD patients and experimental murine colitis compared with healthy controls (176). However, increased receptor expression should be distinguished from evidence of functional involvement in disease. Pharmacological studies indicate that activation of P2Y2 by ATP or UTP promotes migration of human macrophages and neutrophils and can facilitate phagocytic clearance of apoptotic cells and bacteria (14, 177, 178). However, the functional contribution of these mechanisms to IBD pathogenesis in humans remains to be established.

P2Y2 receptor also participates in interactions between intestinal epithelial and immune cells. In human and mouse IECs, P2Y2 activation increases intercellular adhesion molecule-1 (ICAM-1) expression, which may facilitate macrophage transepithelial migration and adhesion (179). Conversely, P2Y2 receptors also contribute to mucosal repair and regeneration by promoting prostaglandin E2 (PGE2) release in rat IECs, stabilizing microtubules, and increasing focal adhesion formation during intestinal inflammation (180, 181). Taken together, these findings indicate that the P2Y2 receptor regulates the inflammatory response, immune defense, and tissue repair. However, caution is needed when applying these findings to humans because the data come from both human expression studies and functional studies in mice and rats, and P2Y responses differ between species.

In addition, expression analysis detected P2Y1 and P2Y2 transcripts in 80% and 56%, respectively, of retrogradely labeled murine colonic neurons, indicating that both receptors are expressed in this neuronal population (179). Pharmacological studies in isolated mouse colonic sensory neurons and visceral nociceptor fibers in mouse and human nerve-gut preparations showed that UTP application increased action potential firing of these neurons, consistent with a role for P2Y2 signaling in sensory neuron activation. Similarly, ADP, an agonist of P2Y1, P2Y12, and P2Y13 receptors, increased action potential firing; this response was inhibited by the selective P2Y1 receptor antagonist MRS2500, providing pharmacological evidence for P2Y1 involvement (182). Together, these findings support a role for P2Y1 and P2Y2 receptors in modulating visceral sensory processes.

Evidence from expression analysis in human tissue, alongside mechanistic findings from experimental models, suggests that the P2Y14 receptor may play a role in IBD. In mucosal biopsies from ulcerative colitis (UC) patients, P2Y14 gene expression is elevated and positively correlated with disease severity (183). These observations support a link between P2Y14 and intestinal inflammation in humans, but they do not establish a causal role for the receptor. Subsequently, mechanistic evidence has emerged from experimental models. In mice with DSS-induced colitis, UDP-glucose, a selective P2Y14 agonist, promotes eosinophil accumulation and activation, whereas genetic deletion of P2Y14 and pharmacological inhibition with the selective antagonist PPTN attenuate disease severity (184). These complementary genetic and pharmacological approaches provide stronger evidence of P2Y14’s functional contribution to experimental colitis than expression data alone. However, the extent to which this eosinophil-dependent mechanism operates in human UC remains to be determined.

Data from a recent study indicate that P2Y14 signaling may also influence IEC survival during experimental colitis (185). P2Y14 overexpression was detected in the intestinal epithelium of mice with DSS-induced colitis, and P2Y14 gene deletion in IECs reduced tissue damage, as its deletion limits the transcriptional activity of cAMP-response element-binding protein through the cAMP/PKA axis, which binds to the promoter of Ripk1, inhibiting necroptosis of IECs. Pharmacological inhibition provided additional evidence supporting this mechanism, as both P2Y14 antagonist PPTN and the small-molecule inhibitor HDL-16 attenuated DSS-induced colitis in mice (185). Together, these observations suggest that P2Y14 may influence experimental colitis through at least two distinct mechanisms involving eosinophil-dependent inflammation and IEC necroptosis. However, these data come primarily from murine models; therefore, the therapeutic efficacy of P2Y14 inhibition, as well as the relevance of these specific pathways in IBD patients, has not yet been determined.

#### 5.1.3 Ectonucleotidases in immunoregulatory responses

The ATP-adenosine axis regulates the homeostatic balance of the intestinal neuro-immune environment and acts as an interdependent molecular switch that balances inflammation and tissue homeostasis. Hydrolysis mediated by NTPDase and CD73 determines the states of this switch, regulating not only eATP clearance but also the temporal and spatial transition from P2 receptor-mediated signaling to adenosine receptor signaling. In general, eATP is associated with danger and pro-inflammatory signals, whereas its degradation to adenosine can promote resolution or immunoregulation (160). However, neither pathway is uniformly pro- or anti-inflammatory, as the outcome depends on receptor subtype, cell type, ligand concentration, and the physiopathological context.

The extracellular balance between ATP and adenosine contributes to immune regulation, and its disruption contributes to the immune dysregulation observed in IBD (176). Evidence from murine IBD models indicates that ectonucleotidases may play a role in controlling this ATP-adenosine balance, as enhancing eATP degradation significantly reduces colonic tissue damage (186, 187), whereas ectonucleotidase deletion exacerbates intestinal inflammation (188, 189). Specifically, genetic deletion of NTPDase-2 or NTPDase-3 worsens intestinal inflammation in a colitis model, with greater disease severity in NTPDase-2-deficient mice, which is associated with macrophage polarization toward a pro-inflammatory phenotype (190). NTPDase-2 and NTPDase-3 are predominantly expressed in enteric neurons and EGCs in both mouse and human colonic tissue; NTPDase-2 is detected in submucosal and myenteric plexuses, whereas NTPDase-3 is mainly localized in the myenteric plexus (190, 191). These localization data establish the presence of the ectonucleotidases within the ENS, but their expression alone does not demonstrate a direct functional mechanism. Nevertheless, the genetic loss-of-function data, together with the observed inflammatory phenotypes, support the interpretation that reduced nucleotide hydrolysis within the ENS may increase local eATP availability and thereby alter P2-receptor signaling in neighboring glial and immune cells. In line with this idea, P2X7 receptors on immune cells and P2Y1 receptors on EGCs are plausible downstream targets, as they promote proinflammatory responses and exacerbate IBD (52, 110). Accordingly, in patients with Crohn’s disease, plasma levels of adenosine diphosphate (ADPase), which reflect NTPDase-2 and -3 activity, are significantly reduced compared with healthy controls and correlate with disease activity. These observations suggest that NTPDase-2 and -3 expressed on ENS may mediate neuroimmune crosstalk and could serve as potential biomarkers for IBD activity, although the human data are primarily associative and do not establish causality.

Other ectonucleotidases also modulate intestinal inflammation. Genetic deletion of NTPDase-1 (CD39) exacerbates intestinal inflammation in a murine colitis model (188). Moreover, Treg cells lacking this ectoenzyme exhibit a reduced capacity to suppress experimental colitis in mice (192). In humans, a common single-nucleotide polymorphism associated with reduced NTPDase-1 levels correlates with increased susceptibility to Crohn’s disease (188). Moreover, in patients with IBD, higher NTPDase-1 expression on peripheral blood Treg cells is associated with clinical and endoscopic remission, whereas lower expression is observed during active disease. Similarly, a subset of Th17 cells with suppressive activity (supTh17) express high levels of NTPDase-1 in the blood and lamina propria and have been shown to contribute to extracellular adenosine production in healthy individuals. However, these cells are reduced in patients with Crohn’s disease (193). Although supTh17 cells share regulatory features with conventional Tregs, including NTPDase-1 expression and adenosine-mediated immunosuppression, they retain Th17-associated characteristics, including IL-17 production and expression of Th17 markers such as CCR6, IL-22, and IL-23R (190). Thus, the protective effects of NTPDase-1 may be mediated, at least in part, by both conventional Treg and supTh17 cells, which contribute to maintaining extracellular adenosine production under physiological conditions, although human studies remain largely correlative and do not establish whether altered NTPDase-1 expression is a cause or consequence of intestinal inflammation.

NT5E (CD73) contributes to adenosine formation from AMP and has also been implicated in the regulation of intestinal inflammation. In mice, genetic deletion of CD73 resulted in more severe DSS-induced colitis, increased IL-1β and TNF-α levels in colonic epithelial tissue, reduced tight-junction protein expression, and increased GI permeability, suggesting a protective role for CD73 in maintaining epithelial barrier function (194). Conversely, pharmacological CD73 blockade has been reported to attenuate intestinal inflammation by modulating macrophage differentiation in mice with DSS-induced colitis (195). In humans, CD73 expression is increased in the colonic mucosa of patients with UC (195), and CD73 expression on CD4^+^ T cells in active IBD is associated with a pro-inflammatory Th17 phenotype (196). These apparently opposing effects of CD73 deficiency and pharmacological blockade may reflect differences in cellular targets, experimental models, disease stage, or intervention timing. Thus, CD73 activity determines extracellular adenosine production, but the consequences of increased adenosine depend on cell-type targets and which adenosine receptors are engaged. Therefore, the discrepancy between global genetic deletion, pharmacological inhibition, and human expression studies underscores that CD73 expression should not be interpreted as a specific biological effect.

#### 5.1.4 Adenosine receptors in immunoregulatory responses

Adenosine receptors are widely expressed on intestinal immune cells, including macrophages, neutrophils, dendritic cells, NK cells, fs, and T cell subsets (Th1, Th17, Th2, and Treg) of humans, mice, and rats (176), as well as on human IECs, enteric neurons, and EGCs (197). While the role of A1 receptors in immune cells remains unclear, substantial evidence supports the immunomodulatory functions of A2A, A2B, and A3 receptors (19).

A2A receptors are expressed at higher levels in human CD4^+^ than CD8^+^ T cells, although expression increases in CD8^+^ cells upon activation (198). In colonic mucosa tissue from patients with UC, miR-16 negatively regulated A2A expression at the post-transcriptional level. Furthermore, transfection of miR-16 mimics promoted nuclear translocation of NF-kappaB p65 protein and expression of pro-inflammatory cytokines, IFN-γ and IL-8, in the human colonic epithelial cell line HT-29, suggesting that miR-16 regulated immune and inflammatory response, at least in part, by suppressing A2A receptor expression (199). A2A receptor activation in mouse T cells inhibits proinflammatory cytokine release, reduces cytotoxic activity (200), and promotes Treg cell expansion with enhanced immunosuppressive capacity (201). Consistent with these findings, A2A-deficient mice show increased susceptibility to inflammatory tissue damage (202), and reduced A2A expression in the GI tract of aged mice is associated with increased inflammation and impaired response to infections (203, 204). Moreover, pharmacological studies provide additional functional support for the protective role of the A2A receptor; administration of the selective agonist ATL-146e reduces intestinal inflammation in murine IBD models by decreasing leukocyte infiltration and proinflammatory cytokine production (205). Similarly, ADA inhibitors exert beneficial effects by enhancing activation of A2A and A3 receptors in rat chronic colitis models (206). Treatment with polydeoxyribonucleotide (PDRN), an A2A agonist, restores intestinal tissue integrity in a rat colitis model by reducing inflammatory cytokine expression and oxidative stress markers; coadministration of the A2A antagonist DMPX abolishes these effects, confirming receptor-specific activity (207). Functional evidence in animal colitis models supports an immunosuppressive role of the A2A receptor; however, human studies currently provide mainly expression and association data. Furthermore, despite extensive evidence supporting its immunosuppressive role in experimental colitis, some studies suggest different effects in other pathological contexts. For example, increased A2A expression during *Trichinella spiralis* infection in mice has been associated with enhanced Th17 polarization and post-infectious IBS-like pathology (208). Thus, the effects of A2A receptor activation must be characterized functionally by cell type, species, and pathophysiological conditions.

The A2A receptor also regulates intestinal contractility. In rat colonic longitudinal muscle preparations, an A2A receptor agonist reduces electrically induced contractile responses, with a greater effect in inflamed tissue, and this response is abolished by an A2A antagonist without affecting carbachol-induced contractions (209). In contrast, in inflamed jejunal and ileal preparations from rats, a selective non-absorbable A2A agonist improves ACh-induced contractions (210) and prevents inflammation-associated contractile dysfunction (211). These apparently discordant findings may reflect differences in intestinal segment, inflammatory state, preparation differences, and neuronal versus smooth muscle receptor localization.

Contrary to the anti-inflammatory effect of the A2A receptor, substantial evidence supports a pro-inflammatory role for the A2B receptor in intestinal inflammation. In a mouse colitis model, genetic deletion or pharmacological antagonism of A2B reduces disease severity and decreases pro-inflammatory cytokine levels (36, 37). Similarly, A2B blockade improves intestinal epithelial integrity and barrier function in models of ischemia-reperfusion injury (IRI) and acute hypoxia in mice (212), reinforcing the idea that A2B activation contributes to epithelial damage and intestinal inflammation. However, A2B receptor signaling may exert protective effects in specific cellular contexts. In mouse IECs, increased A2B receptor signaling attenuates intestinal inflammation (213), possibly through coordinated inhibition of adenosine uptake via the equilibrative nucleoside transporter 2 (ENT2) (214). A2B activation in IECs is also essential for initiating type 2 immune responses against helminths by promoting memory T cell responses (215). Additionally, studies in mice with partial netrin-1 deficiency indicate that A2B receptors contribute to protective pathways regulating leukocyte trafficking during colitis (216). A2B receptors in EGCs appear to represent another compartment with distinct effects. In mice, EGC-specific A2B receptor deletion protects against barrier dysfunction during acute DSS-induced colitis by increasing tight junction protein expression and reducing inflammatory mediators (217). During exposure to *Clostridioides difficile* toxins, A2B expression is reduced in EGCs, whereas A2A and A3 expression increases. Under these conditions, A2A and A3 agonism reduce glial apoptosis, whereas A2B blockade decreases EGC apoptosis, highlighting the coordinated roles of adenosine receptor subtypes in glial cells (218). Collectively, these findings show that the function of adenosine receptors cannot be inferred solely from the expressed receptor subtype levels, but rather depends on their cellular localization and disease context.

A3 receptor signaling is also dependent on cellular and experimental context. In human IECs, A3 receptor activation suppresses NF-κB signaling and reduces pro-inflammatory cytokine production, providing direct functional evidence for an epithelial anti-inflammatory effect (219, 220). Consistent with this observation, A3 activation suppresses cytokine expression via NF-κB inhibition in a DSS-induced colitis model in mice (221). Pharmacological studies further support this role, as treatment with the selective A3 agonist N (6)-(3-iodobenzyl)-adenosine-5-N-methyluronamide (IB-MECA) reduces intestinal inflammation and improves disease outcomes in experimental colitis (222, 223). Similar results have been observed in a rat model of 2,4-dinitrobenzenesulfonic acid (DNBS)-induced colitis, in which the selective agonist AR170 decreases inflammatory cell infiltration, cytokines, and visceral hypersensitivity (224). In 2,4-trinitrobenzenesulfonic acid (TNBS)-colitis mice, electroacupuncture-induced visceral analgesia was associated with upregulation of A3 expression, along with A2A and A1 receptors, suggesting coordinated receptor activation in suppressing inflammation and visceral pain (225). However, these studies primarily demonstrate pharmacological or associative evidence rather than establishing that the A3 receptor is entirely protective.

Human and genetic studies reinforce the complexity of A3 receptor signaling. In colonic epithelial cells from patients with UC, A3 receptor expression is decreased and inversely correlated with miR-206, a microRNA associated with disease activity (226). In murine DSS-induced colitis, increased miR-206 expression correlates with exacerbated inflammation, while inhibition of miR-206 partially restores A3 receptor expression (227). Genetic deletion of A3 protects mice against DSS-induced colitis, reducing CD4^+^ cell infiltration and preserving colonic motility (228). Furthermore, elevated A3R levels in peripheral blood mononuclear cells from patients with rheumatoid arthritis, psoriasis, and Crohn’s disease are associated with activation of NF-κB and CREB signaling (229), suggesting a potential pro-inflammatory role in certain contexts. Overall, pharmacological A3 activation has shown anti-inflammatory effects in human epithelial models and experimental colitis, whereas genetic deletion of the A3 receptor appears protective against DSS-induced colitis in mice. These apparently divergent findings may reflect differences in receptor localization, experimental models, or physiopathological conditions. Consequently, A3 receptor abundance alone does not determine its effect, as this may depend on the cellular context.

Taken together, all these findings support a model in which purinergic signaling orchestrates bidirectional communication among enteric neurons, EGCs, and immune cells. During tissue injury and inflammation, excessive purine release activates multiple purinergic receptors, amplifying neuroimmune purinergic signaling and contributing to epithelial barrier dysfunction, neuroinflammation, altered GI motility, and chronic intestinal inflammation (**Figure 1**).

**Figure 1:**
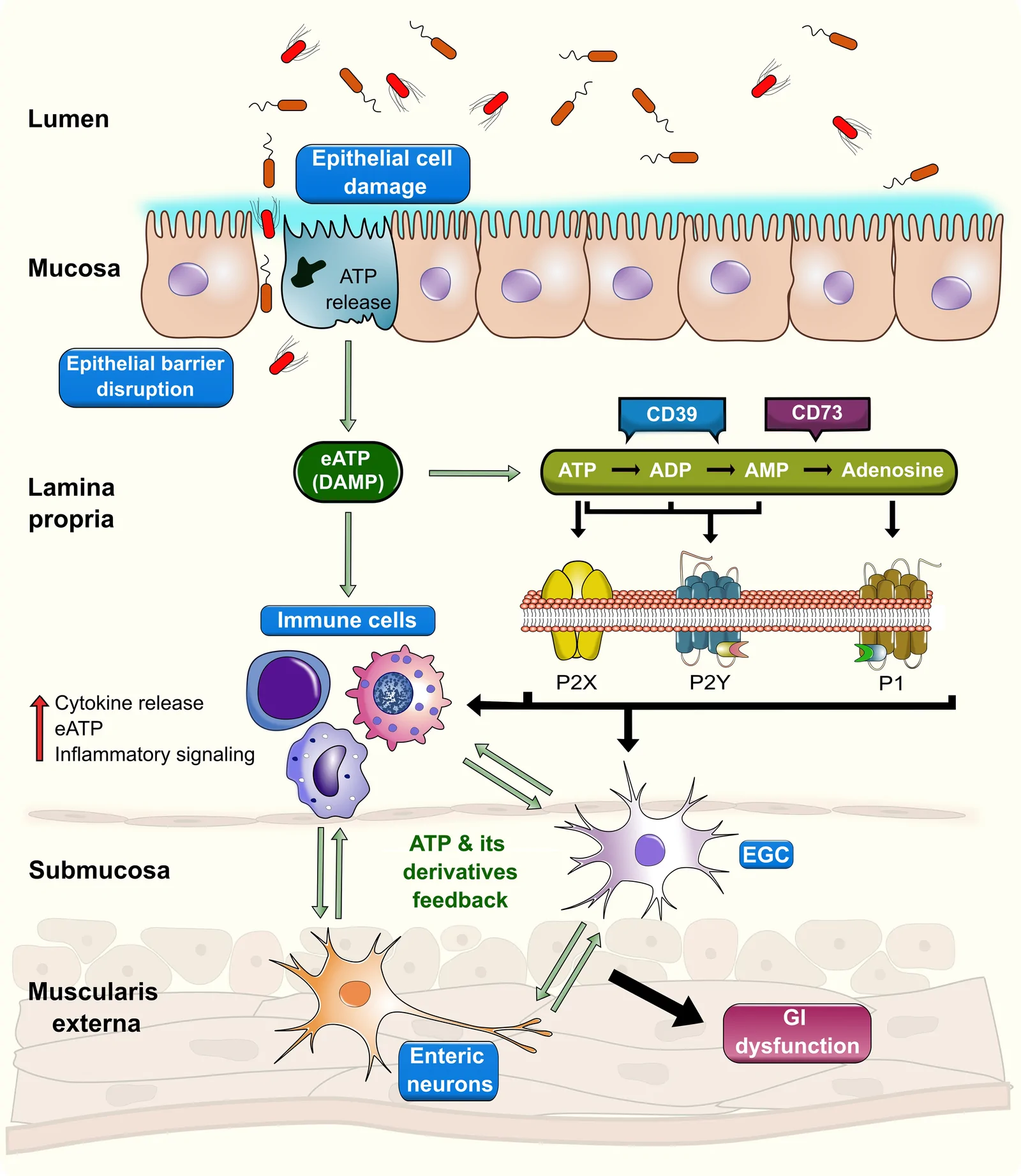
Schematic representation of purinergic signaling-mediated neuroimmune crosstalk in the gut during tissue injury. Epithelial cell damage in the intestine triggers the release of extracellular ATP (eATP), which acts as a danger-associated molecular pattern (DAMP) and activates resident immune cells in the lamina propria, resulting in an inflammatory response that increases cytokine production and additional eATP release. Sequential hydrolysis of ATP to ADP, AMP, and adenosine by the ectonucleotidases CD39 and CD73 modulates ligand availability for P2X, P2Y, and P1 receptors. Purinergic receptor activation promotes a tripartite communication between immune cells, enteric neurons, and EGCs, amplifying pathways that underlie epithelial barrier dysfunction, neuroinflammation, and altered GI motility.

## 6 Discussion and perspective

Current evidence suggests that the purinergic system can serve as a molecular signal for communication among enteric neurons, EGCs, IECs, and immune cells throughout the GI tract. Through the dynamic interplay between eATP, its metabolites, ectonucleotidases, and purinergic receptors, this signaling system regulates intestinal motility, epithelial secretion, barrier integrity, immune surveillance, and tissue repair.

Precise regulation of extracellular nucleotide and nucleoside concentrations contributes to intestinal homeostasis. In contrast, excessive ATP release or altered purine metabolism during infection, tissue injury, or chronic inflammation can disrupt this balance, leading to sustained activation of pro-inflammatory pathways, enteric neuronal dysfunction, and impaired GI motility. Ectonucleotidases regulate ATP and adenosine availability and, consequently, purinergic receptor activation. Therefore, the biological response to purinergic signaling depends not only on the presence of a specific nucleotide or receptor but also on the balance between P2 and P1 receptor signaling, determined by the concentration and cellular source of nucleotide release, the local ectonucleotidase profile, receptor subtype and localization, cell identity, and the disease context. The interplay between these factors could help explain divergent findings. For example, signaling via the same receptor may protect epithelial cells but promote inflammatory or dysfunctional responses in neurons or immune cells. Similarly, a shift from ATP-dominated to adenosine-dominated signaling does not necessarily imply a transition from a pro-inflammatory to anti-inflammatory response. The ultimate effect depends on the activated receptor subtype as well as the characteristics of the cell and the pathophysiological environment in which the signaling occurs.

The ENS constitutes an interface between intestinal physiology and immunity. Ectonucleotidases, such as NTPDase-2 and NTPDase-3, are abundant in enteric neurons and EGCs, enabling the regulation of the local availability and duration of extracellular nucleotides within the ENS. Purinergic signaling in enteric neurons contributes to neurotransmission and intestinal motility, whereas signaling in EGCs can influence the release of inflammatory mediators, epithelial barrier function, and neuro-immune communication. Experimental evidence also suggests that the neuronal release of nucleotides may play an active role in neuro-immune communication. However, this evidence stems primarily from experimental models; it remains unknown whether the same mechanisms operate in humans.

The fact that purinergic receptors and ectonucleotidases are expressed at certain levels does not prove they have a specific function. Regarding intestinal inflammation, the most compelling evidence for their involvement comes from pharmacological and genetic studies in animal models; meanwhile, human studies offer important evidence regarding altered expression of these purinergic receptors and ectonucleotidases in IBD. Yet these observations are associative and therefore cannot establish whether the observed changes are causes, consequences, or adaptive responses to inflammation. Moreover, differences between species and the limitations of pharmacological studies, such as lack of complete selectivity and off-target effects, hinder the direct extrapolation of preclinical findings to humans. Even though genetic models provide stronger evidence of causality, they do not fully mimic the complexity of human disease. Therefore, the contribution of purinergic signaling to GI diseases and its therapeutic relevance must be interpreted with caution.

These considerations are particularly relevant to therapeutic development. Purinergic signaling is involved not only in inflammation but also in physiological processes such as intestinal motility, epithelial secretion, barrier maintenance, sensory signaling, immune surveillance, and host defense. Consequently, systemic inhibition or activation of a purinergic receptor could lead to adverse effects. For example, interfering with P2 or P1 receptor signaling could alter enteric neurotransmission and gastrointestinal motility, epithelial ion transport, mucosal integrity, or antimicrobial immune responses. Furthermore, the cell-type-dependent effects of these receptors raise concerns that systemic receptor modulation might suppress detrimental signals while simultaneously disrupting protective responses in other cell populations. Consequently, therapeutic strategies should prioritize receptor selectivity and, whenever possible, targeted delivery to specific tissues or cells.

Despite substantial advances, several important questions remain to be addressed. Further research is needed to elucidate the precise contribution of each purinergic receptor to communication among neurons, glia, and the immune system, as well as their interactions with gut microbiota and their roles across the various stages and types of GI diseases. Approaches combining single-cell and spatial transcriptomics, spatial proteomic profiling, functional imaging, cell-specific genetic manipulation, and validation in human intestinal tissues could help distinguish between receptor expression and endogenous function. Comparative studies between human tissues and experimental animal models will be crucial for determining which mechanisms are conserved and which may be species-specific. Furthermore, a better understanding of purinergic signaling within the gut neuro-glial-immune axis may identify novel biomarkers of disease activity and therapeutic strategies for IBD, post-inflammatory dysmotility, visceral pain syndromes, and other GI disorders.

Current evidence supports purinergic signaling as a regulatory network connecting enteric neurons, EGCs, epithelial cells, and immune cells. However, its effects are highly context-dependent, and evidence strength varies by receptor and cell type, species, and experimental approach. Although preclinical studies identify some promising mechanisms and potential therapeutic targets, further studies focused on specific cell types and rigorous validation in human tissues and clinical settings are needed to determine which components of the purinergic network drive GI diseases and which represent adaptive responses to inflammation. Therefore, translational progress will depend on demonstrating that modulating a specific purinergic pathway yields a beneficial effect in the appropriate human cell types without impairing essential intestinal functions.
